# DNA aneuploidy relationship with patient age and tobacco smoke in OPMDs/OSCCs

**DOI:** 10.1371/journal.pone.0184425

**Published:** 2017-09-06

**Authors:** Patrizio Castagnola, Sergio Gandolfo, Davide Malacarne, Cinzia Aiello, Roberto Marino, Gabriele Zoppoli, Alberto Ballestrero, Walter Giaretti, Monica Pentenero

**Affiliations:** 1 Department of Integrated Oncological Therapies, IRCCS AOU - San Martino - IST, Genoa, Italy; 2 Department of Oncology, Oral Medicine and Oral Oncology Unit, University of Turin, Turin, Italy; 3 Department of Internal Medicine (Di.M.I.), University of Genoa, Genoa, Italy; Lawrence Berkeley National Lab, UNITED STATES

## Abstract

The aim of this study was to investigate the relationship between tobacco smoke habit, patient age, DNA aneuploidy and genomic DNA copy number aberrations (CNAs) in oral potentially malignant disorder (OPMD) and oral squamous cell carcinoma (OSCC) patients. DNA aneuploidy was detected by high-resolution DNA flow cytometry (hr DNA-FCM) on DAPI stained nuclei obtained from multiple tissue samples from OPMDs/OSCCs in 220 consecutive patients. Nuclear genomic aberrations were determined in a subset of 65 patients by genome-wide array comparative genomic hybridization (aCGH) using DNA extracted from either diploid or aneuploid nuclei suspension sorted by FCM. DNA aneuploidy and mean nuclear genomic aberrations were associated with patients’ age. In particular, DNA aneuploidy strongly associated with age in non-smoker OPMDs/OSCCs patients. OSCCs from smokers showed a lower prevalence of DNA aneuploidy compared to OSCCs from non-smokers. A higher occurrence of DNA aneuploidy (particularly in smokers’ OPMDs) was observed in patients characterized by involvement of a single oral subsite. Our study suggests that: 1) DNA aneuploidy in non-smokers is mainly related to aging; 2) OPMDs/OSCCs involving multiple oral subsites in smokers are less likely to develop DNA aneuploidy compared to non-smokers; 3) OSCC development is characterized by both CIN and CIN-independent mechanisms and that the latter are more relevant in smokers. This study provides evidence that DNA diploid OPMDs may be considered at lower risk of cancerization than DNA aneuploid ones in non-smokers but not in smokers.

## Introduction

Oral potentially malignant disorders (OPMDs) are an heterogeneous group of asymptomatic mucosal alterations of various etiology associated to alteration of the nuclear genome that may lead to oral squamous cell carcinomas (OSCCs) [1]. Estimates of the transformation rate of OPMDs in the literature vary [2] but there is a wide consensus on a frequency of about 1.36% per year and 95% confidence intervals (CI) between 0.69 and 2.03% [3]. To date, the histological detection of dysplasia is the most important criterion followed by oral surgeons to decide the excision of OPMDs in an effort to prevent OSCCs [4]. However, no clinical studies that prove the efficacy of this approach have been reported so far. Several studies were conducted in an effort to find additional criteria that could predict the progression to OSCC of a given OPMD. Clinical studies showed that some subsites of the oral mucosa, which included tongue and floor of the mouth, have an increased risk of cancer development [5]. Others found a relationship of DNA aneuploidy, which reflects chromosomal instability (CIN), with dysplasia [6–11]. Further studies provided also evidences of an association between DNA aneuploidy with high-risk oral mucosa subsites [12] and with genomic copy number aberrations [13]. However, the proof that DNA aneuploidy can predict the progression of given OPMD in a clinical setting is still missing and the same applies for some molecular markers, such as p53 (TP53), Cyclin D1, and podoplanin (PDPN), HIF-1alpha, E-cadherin, and p63, which were also investigated [14, 15].

Tobacco consumption, either chewed or smoked, is recognized as the major risk factor for OPMDs and OSCCs [16] and is associated to cytotoxic and genotoxic effects [17]. Tobacco smoke, in particular, contains carcinogens that bind to the DNA generating DNA adducts [18], which in turn may cause mutations able to activate cellular oncogenes or inactivate tumor suppressor genes [19, 20]. Additionally, tobacco components are potent free radical generators, which may cause genotoxic effects by inducing single strand DNA breaks [21]. It was also shown that tobacco use induces an increase in the number of aneuploid nuclei in the oral epithelium [22]. However, a recent study indicates that in the oral epithelium tobacco smoke mainly induces acceleration of cellular mechanisms that in turn lead to genomic DNA damage [23, 24].

The relationship between aging and DNA aneuploidy has been investigated extensively in the scientific literature but, as far as we know, poorly in OPMDs and OSCCs. Our previous study with 60 OPMD patients enrolled, suggested that DNA aneuploidy is an early event in oral carcinogenesis and that the effects of tobacco smoke is age- and oral subsite-dependent [25]. The aim of this study was to test these results on a substantially larger OPMDs/OSCCs patients’ cohort. We took advantage of high resolution DNA flow cytometry (hr DNA-FCM) of DAPI stained nuclei extracted from tissue samples obtained from OPMDs/OSCCs to determine the DNA Index (DI) and used logistic regression to evaluate the role of each variable considered in the study.

## Materials and methods

### Patients and tissue specimens

Patients with OPMDs or OSCCs were enrolled in the study by the Oral Medicine and Oral Oncology Unit of the University of Turin at the A.O.U. S. Luigi Gonzaga (Orbassano-Turin). Written informed consent was obtained from all the enrolled patients as requested by the Institutional Ethics Committees (A.O.U. S. Luigi Gonzaga Prot. N. 11780), which specifically approved this study. Protocols included in the Declaration of Helsinki were followed in designing the study.

Tobacco smoke and alcohol consumption habit were recorded during the interview that was performed to inform the patient on the aim of the study and to collect the patient’s consent to participate.

Histological evidence of one or more OPMDs (homogeneous and non-homogeneous leukoplakias, erythroplakias and erythroleukoplakias) or of OSCC was considered inclusion criteria.

Tissue samples for histological diagnosis and hr DNA-FCM were obtained from each OPMD/OSCC by incisional biopsies and micro-biopsies (carried out by means of a curette) as previously reported [26]. Tissue samples were also obtained by microbiopsies from mucosa at a distance from co-existing OPMDs/OSCCs and characterized by visually normal appearing mucosa. All patients were characterized by the presence of at least one OPMD/OSCC.

Patients with history of previous oropharyngeal cancer diagnosis were excluded from the study.

Histological diagnosis was performed according to WHO guidelines by a specially trained pathologist [26, 27].

Tissue samples for hr DNA-FCM analysis were either immediately processed or stored at -20°C and processed at a later time.

### Nuclei extraction from oral microbiopsies, DAPI staining and hr DNA-FCM analysis

DAPI (4',6-diamidino-2-phenylindole) stained nuclei suspensions from tissue samples were obtained as described by Otto et al. [28] with previously reported modifications [29]. DNA content histograms were generated from DAPI stained nuclei suspensions by hr DNA-FCM performed as previously reported [29] and analyzed to evaluate the DNA Index (DI).

When DNA aneuploid sublines (DI ≠ 1) were detected, these were sorted using a Cyflow Space FCM equipped with a PPCS unit (Partec GmbH, Muenster, Germany) at a purity of about 99%.

Normal oral mucosae obtained from 3 healthy individuals used as normal controls showed a DI = 1. In S1 Fig, we show the DNA content histograms as determined by hr DNA-FCM analysis of these normal controls compared with 3 OPMDs/OSCCs samples with DI≠1.

Representative pathological tissue sections for OPMD and OSCC are shown in S2 Fig.

### DNA copy number analysis by aCGH

aCGH files were processed as described previously [13]. In brief, Agilent feature extraction files were parsed in R, assessed for derivative log ratio spread (dLRS) using the function available in the supplementary material, and converted in to *log2* ratio space. In general, files with a dLRS > 0.35 were discarded, although we retained or discarded some samples based on visual inspection of the karyogram plots. Probes were averaged over replicates, and used as input for segmentation as follows: after probe value winsorization, data were segmented using the *pcf* or the *multipcf* function available in the R/BioConductor package copynumber with default settings [30]. Segments were considered aberrant if their *log2* ratio values were above 0.32 for copy gains and below -0.41 for copy losses, based on the assumption of a minimum cancer cell fraction of 50% in the analyzed specimens and a mostly clonal population. For circular karyograms and genome plots, we used the respective functions available in the copynumber package. To infer a measure of genomic instability for each patient, we first summed the number of aberrant segments for each sample from each patient. Since multiple samples were subjected to aCGH analysis for each patient, the average of aberrations per sample was used for further analysis. We used the Fisher exact test for count data to assess whether genomic instability was associated with ageing as a categorical parameter (older or younger than median) and smoking. To assess whether statistically significant results were independently associated with genomic instability, we fitted a multivariable linear model with the number of aberrations per patients as a dependent variable, age, and smoking as categorical dependent variables. The interaction of age and smoking with genomic instability was not modeled due to the relative small sample size of our set. P values were all calculated using two-sided tests. All statistics were performed in the R environment for statistical computing and graphics. Metadata from the 65 patients included in this aCGH study are available in S1 File. Raw and processed data from OPMDs/OSCCs patients’ samples are available in GEO (http://www.ncbi.nlm.nih.gov/geo/) under the accession number GSE66136.

### Statistical analysis

Binary logistic regression analysis was performed to explore the influence of various patients’ characteristics on the dichotomous outcome, which is a DNA diploid (DI = 1) or a DNA aneuploid status (DI≠1).

When indicated, the two-tailed Mann–Whitney (MW) U test was applied to test differences in age distribution between two independent groups of patients while the Kruskal-Wallis test was applied to test differences in age distribution among more than two independent groups of patients. To correct for multiple comparison, false discovery rates (FDR) q-values were calculated as previously reported [31].

Current and former tobacco smokers’ patients were both included in the smokers’ patients subgroup while the non-smokers’ subgroup included patients that never smoked.

Former and current drinkers were both included in the drinkers’ subgroup while occasional drinkers were included with abstainers in the non-drinkers’ subgroup.

Patients with OPMDs in multiple oral subsites were represented by the OPMD with the most severe histological diagnosis.

## Results

### Patients’ characteristics age distribution among patient subgroups

Patients’ characteristics are shown in Tables 1–3 and differences in age distribution among patient subgroups are shown in Table 1.

**Table 1:** Patients’ characteristics and differences in age distribution among patient subgroups.

Patients’ data	Number	Median age in years (range)	
Male	100	62.1 (18.5–87.8)	P = 0.002^a^
Female	120	67.4 (19.8–93.8)	
Non-smokers	91	69.4 (18.5–93.8)	P = 0.002^a^
Smokers	129	62.9 (19.8–87.8)	
ND-OPMDs^b^	126	63.1 (26.2–92.8)	P = 0.004^c^
D-OPMDs^d^	26 (17 mild, 7 moderate, 2 severe)	65.7 (18.5–93.8)	
OSCCs^e^	68	71.0 (19.8–90.6)	
Single oral subsite OPMDs/OSCCs	158	66.5 (19.8–93.8)	P = 0.352^a^
Multiple oral subsite OPMDs/OSCCs	62	64.5 (18.5–87.3)	
DNA diploid	125	62.9 (18.5–89.8)	P = 0.0001^a^
DNA aneuploid	95	70.6 (19.8–93.8)	

^a^Mann–Whitney (MW) U test.

^b^Non dysplastic oral potentially malignant disorders, ND-OPMDs.

^c^Kruskal-Wallis test.

^d^Dysplastic oral potentially malignant disorders, D-OPMDs.

^e^Oral squamous cell carcinomas, OSCCs.

**Table 2:** Patients’ tobacco consumption details (54 former and 76 current smokers).

	Tobacco smoke free (in former smokers; months)	Cigarette packs/day	Cigarette packs/year	Smoke duration (years)
Median	180	1	24	35
Minimum	2	1/20	1.5	3
Maximum	600	3	120	70

**Table 3:** Patients’ alcohol consumption details.

	Alcohol Units/day
Median	1
Minimum	0.5
Maximum	8

### Relationship between ploidy status, age, and smoke habit, in OPMDs^a^ and OSCCs^b^ patients

The relationship of DNA aneuploiy (DI ≠ 1), in all OPMDs/OSCCs, with age and tobacco smoke habit, was analyzed by logistic regression. The presence or absence of DNA aneuploidy was the response variable for the logistic regression. Age was taken as a covariate variable in the model.

The risk of occurrence of DNA aneuploidy in OPMDs/OSCCs with each year increase in age was 1.03 (P = 0.027) (Table 4).

**Table 4:** Relationship between ploidy status, age, and smoke habit, in OPMDs^a^ and OSCCs^b^ patients.

DNA aneuploidy in patients with OPMDs or OSCCs	Odds Ratio	P-value^c^	Lower 95% Confidence interval	Upper 95% Confidence interval
**All patients**				
Age	1.03	**0.027**	1.00	1.05
OSCCs vs OPMDs	8.28	**<0.0005**	4.01	17.1
Multiple vs single oral subsite OPMDs/OSCCs	0.35	**0.007**	0.16	0.75
Smokers vs Non-smokers	0.73	0.397	0.35	1.51
Drinkers vs Non-drinkers	1.11	0.770	0.56	2.21
Gender (female vs male)	0.78	0.507	0.38	1.62
**OPMD patients**				
Age	1.03	0.051	1.00	1.06
D-OPMDs^d^ vs ND-OPMDs^e^	3.08	**0.017**	1.22	7.76
Multiple vs single oral subsite OPMDs	0.21	**0.008**	0.07	0.67
Smokers vs Non-smokers	1.51	0.391	0.59	3.90
Drinkers vs Non-drinkers	1.19	0.693	0.51	2.78
Gender (female vs male)	0.77	0.568	0.32	1.87
**OSCC patients**				
Age	1.03	0.201	0.98	1.08
Multiple vs single oral subsite OSCCs	0.55	0.391	0.14	2.13
Smokers vs Non-smokers	0.17	**0.021**	0.04	0.76
Drinkers vs Non-drinkers	0.72	0.617	0.20	2.63
Gender (female vs male)	1.03	0.973	0.24	4.39
**Non-smoker patients**				
Age	1.12	**<0.0005**	1.05	1.19
OSCCs vs OPMDs	73.06	**<0.0005**	11.20	476.55
Multiple vs single oral subsite OPMDs/OSCCs	0.28	0.138	0.05	1.50
Drinkers vs Non-drinkers	1.15	0.883	0.19	7.09
Gender (female vs male)	0.41	0.371	0.06	2.93
**Smoker Patients**				
Age	1.00	0.929	0.97	1.03
OSCCs vs OPMDs	4.21	**0.002**	1.72	10.27
Multiple vs single oral subsite OPMDs/OSCCs	0.24	**0.005**	0.09	0.65
Drinkers vs Non-drinkers	0.80	0.578	0.36	1.76
Gender (female vs male)	0.70	0.399	0.30	1.61

^a^Oral potentially malignant disorders, OPMDs.

^b^Oral squamous cell carcinomas, OSCCs.

^c^P- values <0.05 are in bold.

^d^Dysplastic oral potentially malignant disorders, D-OPMDs.

^e^Non dysplastic oral potentially malignant disorders, ND-OPMDs.

When we examined the OPMDs/OSCCs from the non-smoker patients’ subgroup, the OR of age increased to 1.12 (P<0.0005) (Table 4). On the contrary, for the smokers’ subgroup, we could not find a significant association of DNA aneuploidy with age (OR 1.00, 95%CI 0.97–1.03). Accordingly, the difference in ORs of age, between non-smokers and smokers, resulted highly statistically significant (P = 0.006, Z test for OR difference).

According to Holgersson and coworkers [32], we preferred to run separate models for smoker and non-smoker groups. In this case, the variance across the two different category analyses is not a priori known to be homogeneous, and category-wise approach is invariant to a possible difference in-group variances. However, the interaction term also reached statistical significance for age and smoking status (OR = 1.097, P = 0.001), when added to the logistic regression model performed on the “all patients” group.

**Fig 1:**
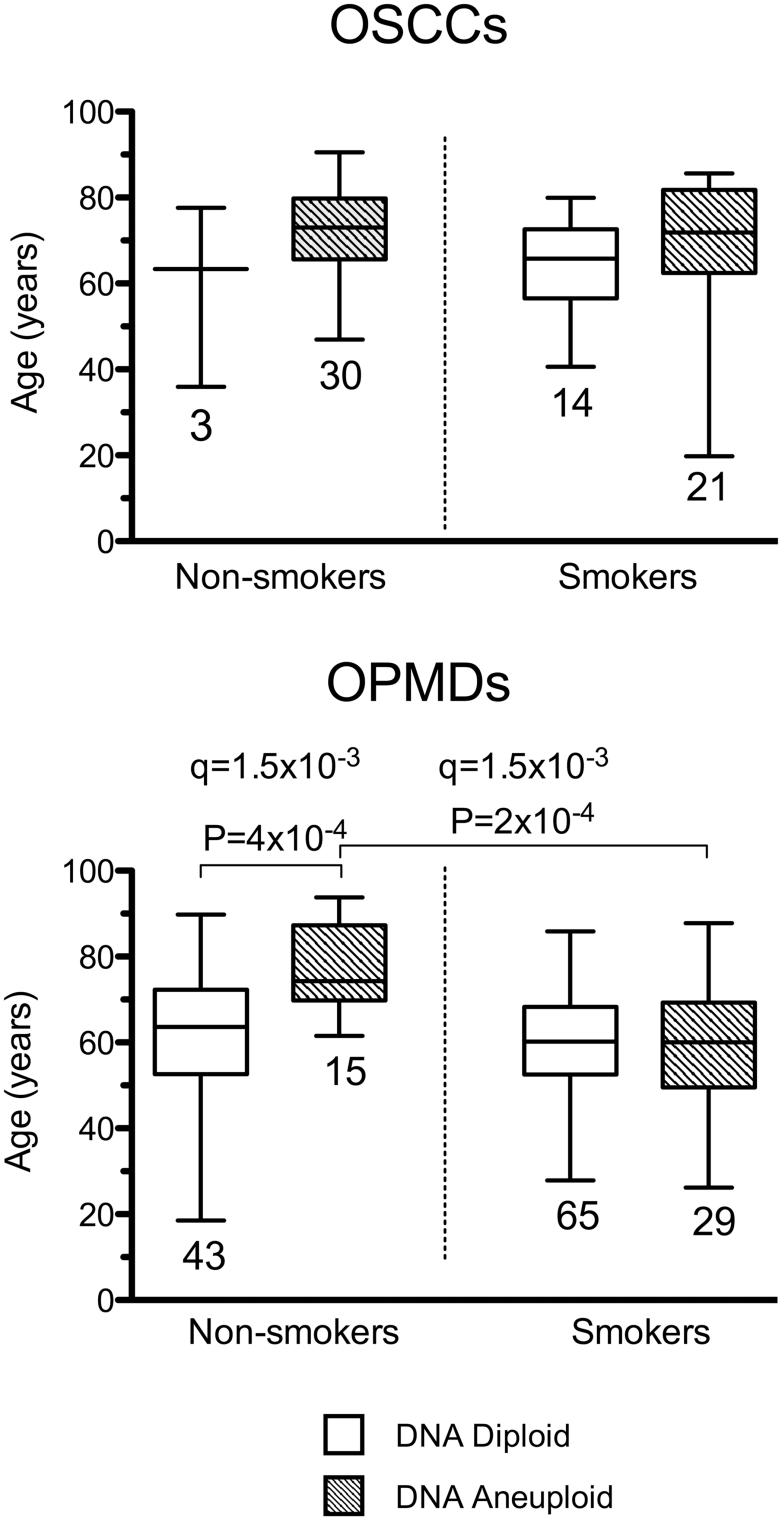
Age distribution, tobacco smoke habit and DNA ploidy status in oral potentially malignant disordes (OPMDs) and oral squamous cell carcinomas (OSCCs) patients. The bottom and the top of each box show the first and third quartile while the line inside the box represents the median (second quartile). The tips of the whiskers represent the minimum and the maximum data value. The number of patients for each category is indicated at the bottom of the corresponding box. The boxes corresponding to DNA diploid OPMDs/OSCCs are white while those corresponding to DNA aneuploid OPMDs/OSCCs have a striped pattern. Significant (MW test) P-values (P < 0.05) are indicated. The FDR q-value method was applied for multiple testing (n = 8) correction and the resulting q-values are indicated.

It should be noticed that although limiting our logistic regression analysis to ND-OPMD patients, we still found, a significant difference (P = 0.006) between the OR of age in non-smokers (1.13, 95%CI 1.04–1.24) and smokers (0.99, 95%CI 0.96–1.03). On the other hand, in OSCC patients the difference between ORs for age in non-smokers and smokers did not reach statistical significance (OR 1.07, P = 0.158 and OR 1.01, P = 0.674; Z-test P = 0.340). Among non-smokers, we found that the median age of the patients with DNA aneuploid OPMDs (74.31 years) was significantly higher compared to those with DNA diploid OPMDs (63.61 years) (Fig 1).

Notably, we found that smokers with DNA aneuploid OPMDs had a younger median age of 14.23 years (Fig 1) with respect to non-smokers with DNA aneuploid OPMDs (Fig 1). Furthermore, in OSCC patients, we observed that 30 out of 33 (90.9%) OSCCs were DNA aneuploid among the non-smokers compared to only 21 out of 35 (60%) among the smokers (Fig 1). In this case the OR was 0.17 (P = 0.021) as estimated by logistic regression analysis (Table 4).

With regards to the relationship of DNA aneuploidy with histology and smoke habit, as expected from our previous study [13], the odds ratio (OR) of the association of DNA aneuploidy was statistically significant in D-OPMDs vs ND-OPMS and in OSCCs vs OPMDs in our cohort of patients (Table 4). In both the non-smokers’ and smokers’ subgroups the association of DNA aneuploidy with OSCCs was significantly greater than with OPMDs (Table 4). However, this association was significantly higher in the non-smokers’ subgroup (Z test P = 0.007).

In this study we also investigated the relationship between DNA aneuploidy, smoke and the presence of OPMDs involving single or multiple anatomical subsite of the patients’ oral mucosa. In our cohort of patients, in the OPMDs and in the smokers’ subgroup the OPMDs involving a single subsite were significantly associated to DNA aneuploidy (P = 0.008 and P = 0.002, respectively) (Table 4).

We also investigated the relationship between alcohol consumption and aneuploidy in our dataset and did not find a statistically significant association (Table 4). Similarly, the gender was never a significant confounding factor (Table 4).

### Relationship between genomic aberrations, age, and smoke habit, in OPMDs and OSCCs patients

**Fig 2:**
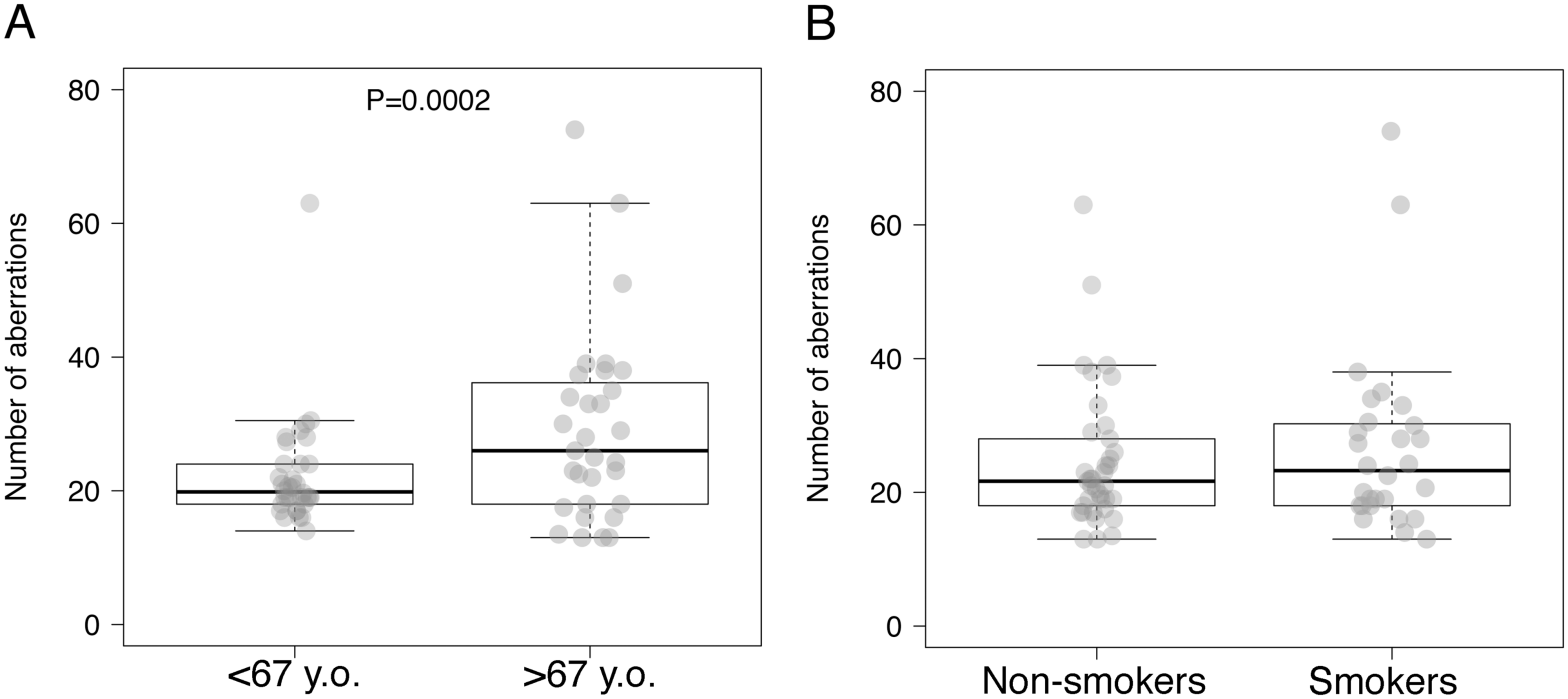
Mean number of genomic aberrations (MNGA) per patient and age or smoking status in oral potentially malignant disordes (OPMDs) and oral squamous cell carcinomas (OSCCs) patients. The average number of aberrations per patient is represented as gray dots superimposed over boxes. These present a thick horizontal line indicating the median number per group, and delimitate the 25^th^ and 75^th^ percentile, while whiskers show the 95% confidence interval. A) average number of aberrations per patients’ age; B) average number of aberrations per patients’ smoking habit.

To assess genomic aberrations and generate virtual karyotypes in our patient set, aCGH analysis was performed on oral mucosa in a subset of 65 patients with OPMDs/OSCCs and in 2 healthy individual used as controls. DNA copy number gains and losses were detected in both smokers and non-smokers OPMDs/OSCCs mucosae (S3 Fig) whereas neither gains nor losses were detected in normal oral mucosa controls (S4 Fig). Furthermore, to assess the relationship between genomic aberrations with age and smoking status, the mean number of genomic aberrations (MNGA) was determined for each subject. We found a statistically significant association between the MNGA per patient with age (22.2 in patients younger than median age vs. 29.2, P = 0.0002, Fig 2A), but not with smoking habit (24.7 in nonsmokers vs. 26.7 in smokers, Fig 2B). The association between MNGA and age remained significant by multiple regression (β coefficient = 7.4, 95%CI = 1.6–13.2, P = 0.0186).

## Discussion

The aim of this study was mainly to assess the relationship between aging, tobacco smoke and DNA aneuploidy, determined by hr DNA-FCM, in OPMDs/OSCCs.

Our results showed a higher occurrence of both DNA aneuploidy and MNGA with patients’ age. In particular, among the non-smokers the association of patients’ age with DNA aneuploid OPMDs/OSCCs was significantly higher compared to those with DNA diploid OPMDs/OSCCs. This is in agreement with our previous study [25]. In addition, this observation was strengthened by the logistic regression analysis, which confirmed the relationship between DNA aneuploidy and age. It should be also noticed that this result did not depend from a different prevalence of OSCCs cases between the two patient subgroups. In fact, in our dataset the number of OSCCs was lower in the non-smoker subgroup and no statistically significantly difference in median age was found between non-smokers and smokers with DNA aneuploid OSCCs. Furthermore, the detected relationship between DNA aneuploidy and age appeared more pronounced in the non-smokers group when the analysis was limited to ND-OPMDs patients.

Overall, our results support the hypothesis that the causes that generate DNA aneuploidy in the oral mucosa are different between non-smokers and smokers. In particular, it appears that aging-related genotoxic mechanisms play a more important role in non-smokers while smoke-related genotoxic damage is an additional factor involved in smokers.

Notably, we could not reveal differences in DNA aneuploidy occurrence between smokers and non-smokers in our entire patient dataset, which appears in line with a recent cytological study performed on normal mucosa [33]. Similarly, our aCGH analysis did not detect differences of MNGA in smokers compared to non-smokers OPMDs/OSCCs.

In the present study the association between DNA aneuploidy and OSCCs was much higher in the non-smokers’ group. This result suggests that while age-related CIN may be the main genetic mechanism that drives cancer development of OPMDs in non-smokers, a substantial amount of OPMDs in smokers progress to cancer via CIN-independent mechanisms, which include gene mutations. In our view this result may have potential application in clinical practice. In particular, DNA diploid OPMDs may be considered at lower risk of cancerization than DNA aneuploid ones in non-smokers but not in smokers.

On the basis of the relationship between DNA aneuploidy and single site OPMDs here reported, we suggest that while in smokers OPMDs involving single oral subsites may progress to cancer through CIN-related mechanisms, those that involve multiple oral subsites may progress mainly through CIN-unrelated mechanisms.

Our study also showed that in our dataset neither alcohol consumption nor gender were confounding factors.

## Conclusions

The present study shows for the first time a significant relationship between aging and DNA aneuploidy in OPMDs and between tobacco smoke and early appearance of DNA aneuploidy in OPMDs. However, as these relationships have an OR close to 1, further studies are required to establish whether they are of wide general validity.

## Supporting information

S1 Fig DNA histograms obtained by high resolution DNA flow cytometry from oral mucosa. X axes show DNA content; Y axes show number of nuclei. A) Subject Ctr1A normal mucosa; B) Subject Ctr2A normal mucosa; C) Subject Ctr7B normal mucosa; D) Pt111B tongue OPMD; E) Pt108B tongue OSCC; F) Pt90B tongue OSCC. DNA Diploid (DI = 1.0) and DNA aneuploid (DI≠1) peaks are indicated.
(TIF)

S2 Fig Representative pathological tissue sections. Non-dysplastic OPMD (A), OPMD with mild-moderate dysplasia (B), OSCC (C).
(TIF)

S3 Fig Circular karyograms obtained from aCGH data. In clockwise direction, the proportion of gained (red) or lost (steel blue) regions is plotted over the corresponding chromosomes. The higher the bar over a given region, the more frequent the aberration. A) Non-smokers; B) Smokers.
(TIF)

S4 Fig Genome plots. Segments (thick red line) are plotted over the individual probe values in log2 ratio space; horizontal black lines represent the threshold for gain (0.32) and loss (-0.41) assuming a fully clonal sample with 50% of cancerous cells and one copy gain or loss. No losses or gains exceeding such cutoffs can be observed in the two healthy control samples represented. Small red stars represent probes considered as outliers after winsorization and not used for segment computation. A) Normal control sample Ctr1A_1; B) normal control sample Ctr7B_1. On the top of each plot the aCGH raw file ID is shown (see GEO Accession Number in Materials and methods).
(TIF)

S1 File Metadata from the 65 patients included in this aCGH study. (XLSX)
