# Supplementary figures and images for: DNA aneuploidy relationship with patient age and tobacco smoke in OPMDs/OSCCs
Source: PLoS One. 2017 Sep 6;12(9):e0184425. doi: 10.1371/journal.pone.0184425 (PMC5587305; doi:10.1371/journal.pone.0184425)

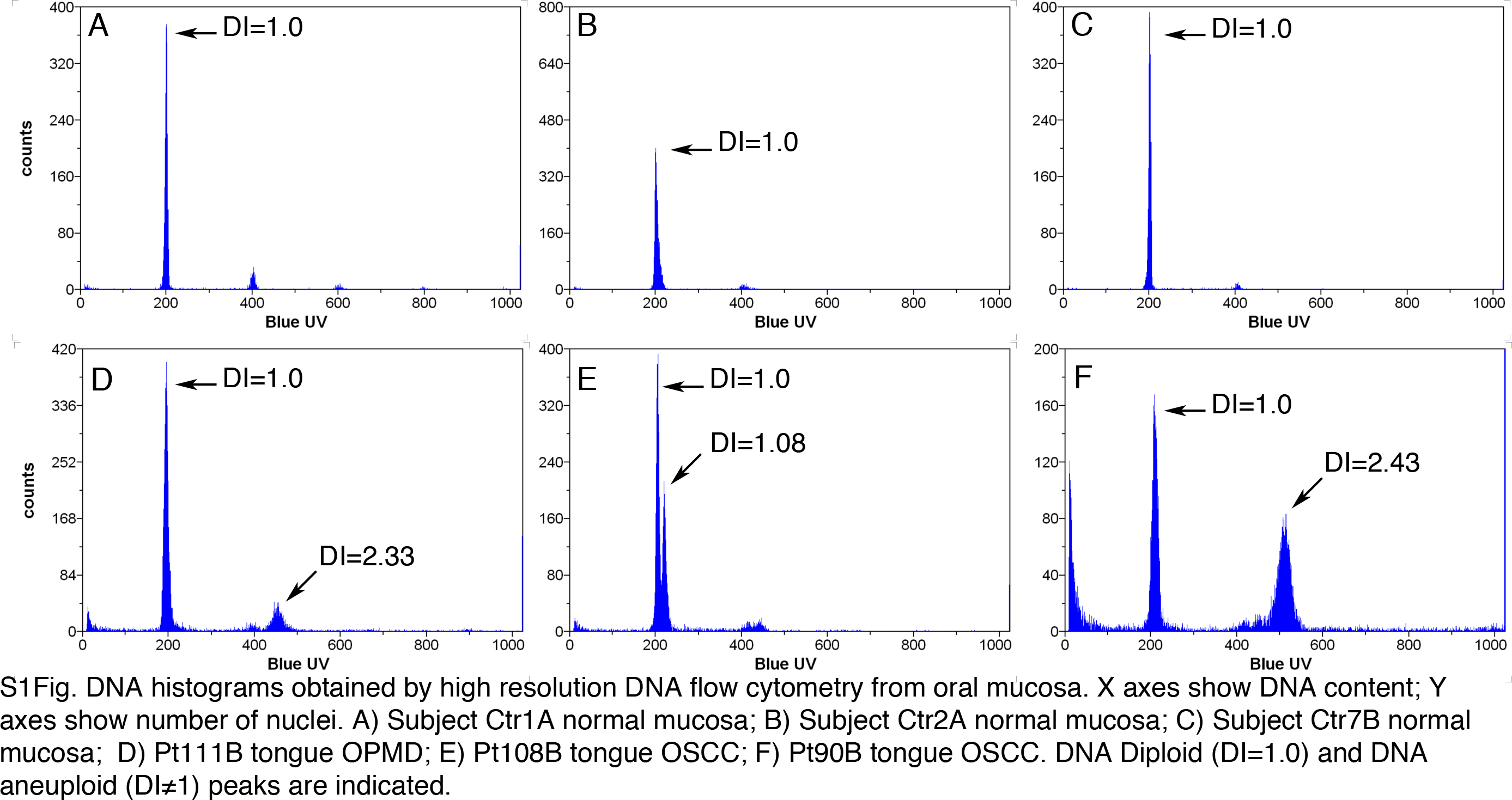

Supplement: S1 Fig — X axes show DNA content; Y axes show number of nuclei. A) Subject Ctr1A normal mucosa; B) Subject Ctr2A normal mucosa; C) Subject Ctr7B normal mucosa; D) Pt111B tongue OPMD; E) Pt108B tongue OSCC; F) Pt90B tongue OSCC. DNA Diploid (DI = 1.0) and DNA aneuploid (DI≠1) peaks are indicated. (TIF) [file pone.0184425.s001.tif]

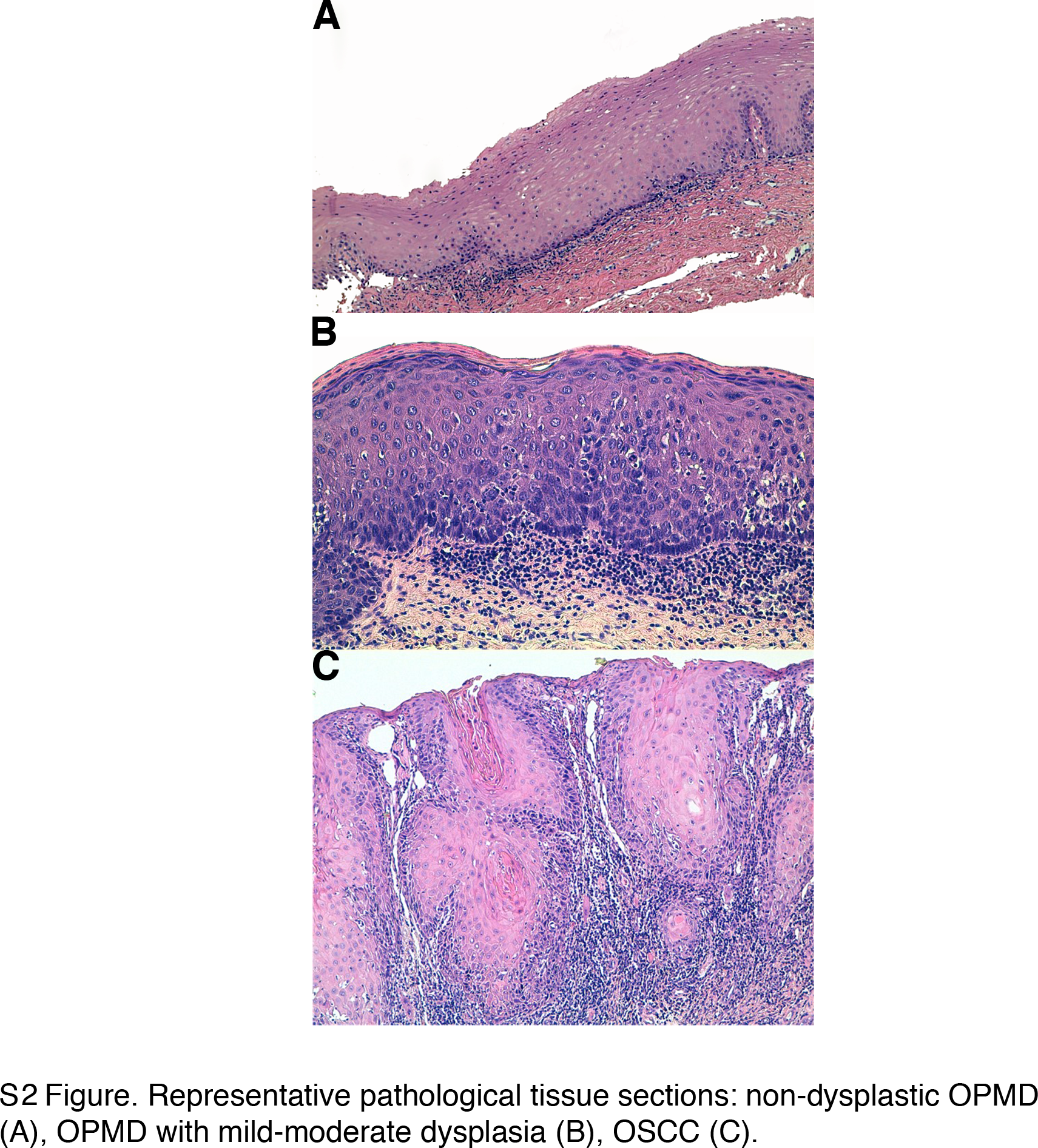

Supplement: S2 Fig — Non-dysplastic OPMD (A), OPMD with mild-moderate dysplasia (B), OSCC (C). (TIF) [file pone.0184425.s002.tif]

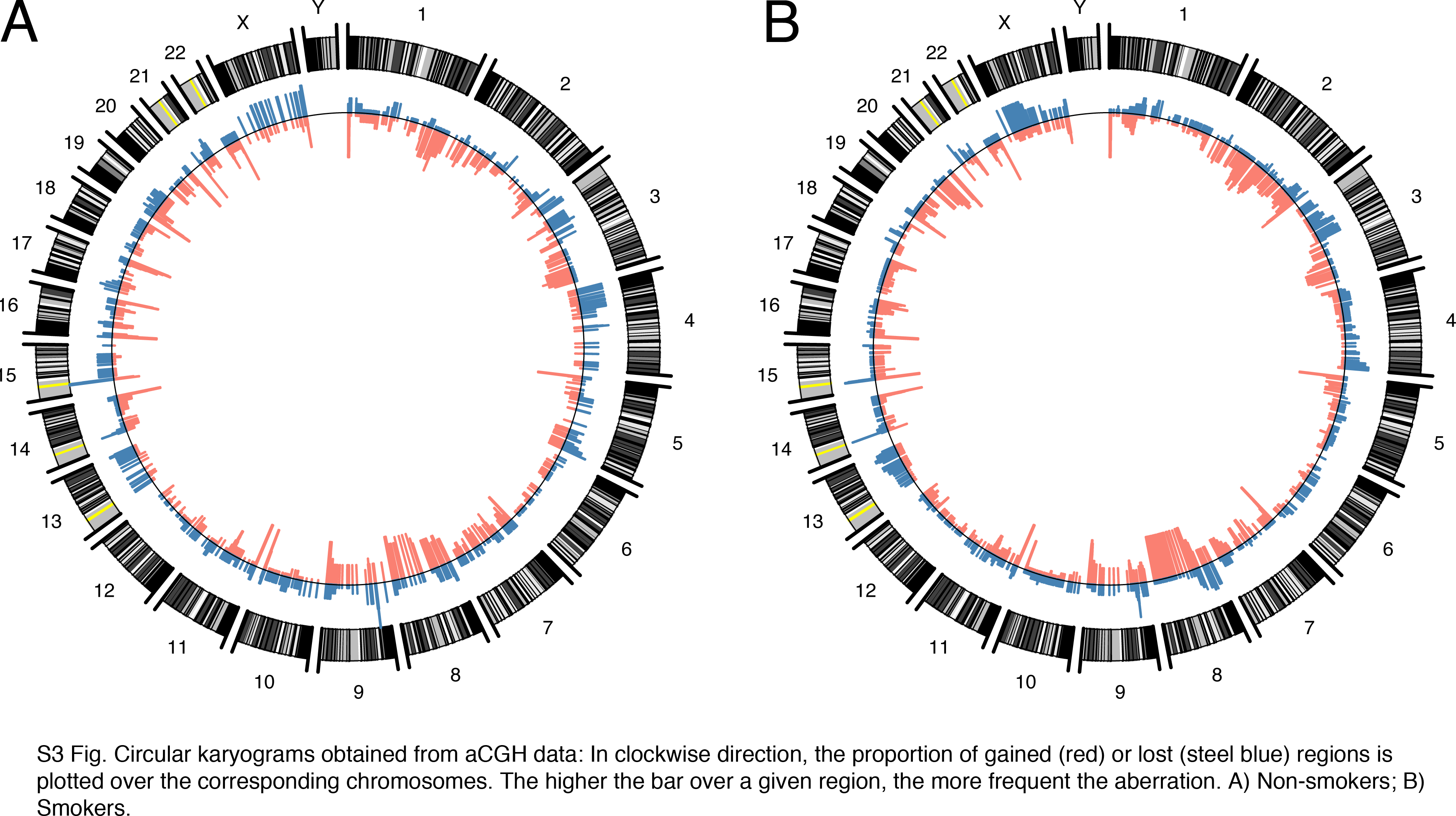

Supplement: S3 Fig — In clockwise direction, the proportion of gained (red) or lost (steel blue) regions is plotted over the corresponding chromosomes. The higher the bar over a given region, the more frequent the aberration. A) Non-smokers; B) Smokers. (TIF) [file pone.0184425.s003.tif]

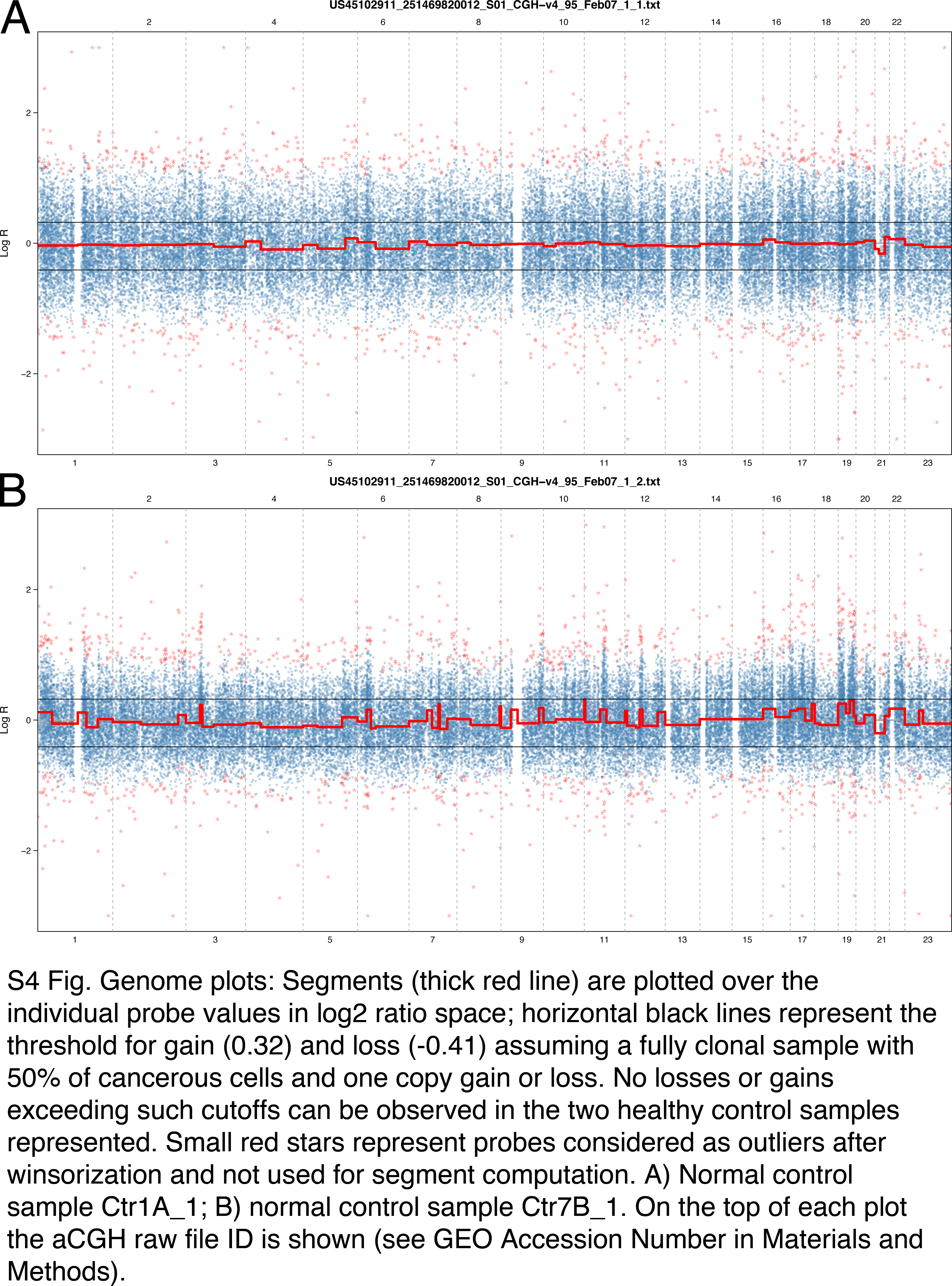

Supplement: S4 Fig — Segments (thick red line) are plotted over the individual probe values in log2 ratio space; horizontal black lines represent the threshold for gain (0.32) and loss (-0.41) assuming a fully clonal sample with 50% of cancerous cells and one copy gain or loss. No losses or gains exceeding such cutoffs can be observed in the two healthy control samples represented. Small red stars represent probes considered as outliers after winsorization and not used for segment computation. A) Normal control sample Ctr1A_1; B) normal control sample Ctr7B_1. On the top of each plot the aCGH raw file ID is shown (see GEO Accession Number in Materials and methods). (TIF) [file pone.0184425.s004.tif]
